# Supplementary figures and images for: Updated Gene Prediction of the Cucumber (9930) Genome through Manual Annotation
Source: Plants (Basel). 2024 Jun 9;13(12):1604. doi: 10.3390/plants13121604 (PMC11207753; doi:10.3390/plants13121604)

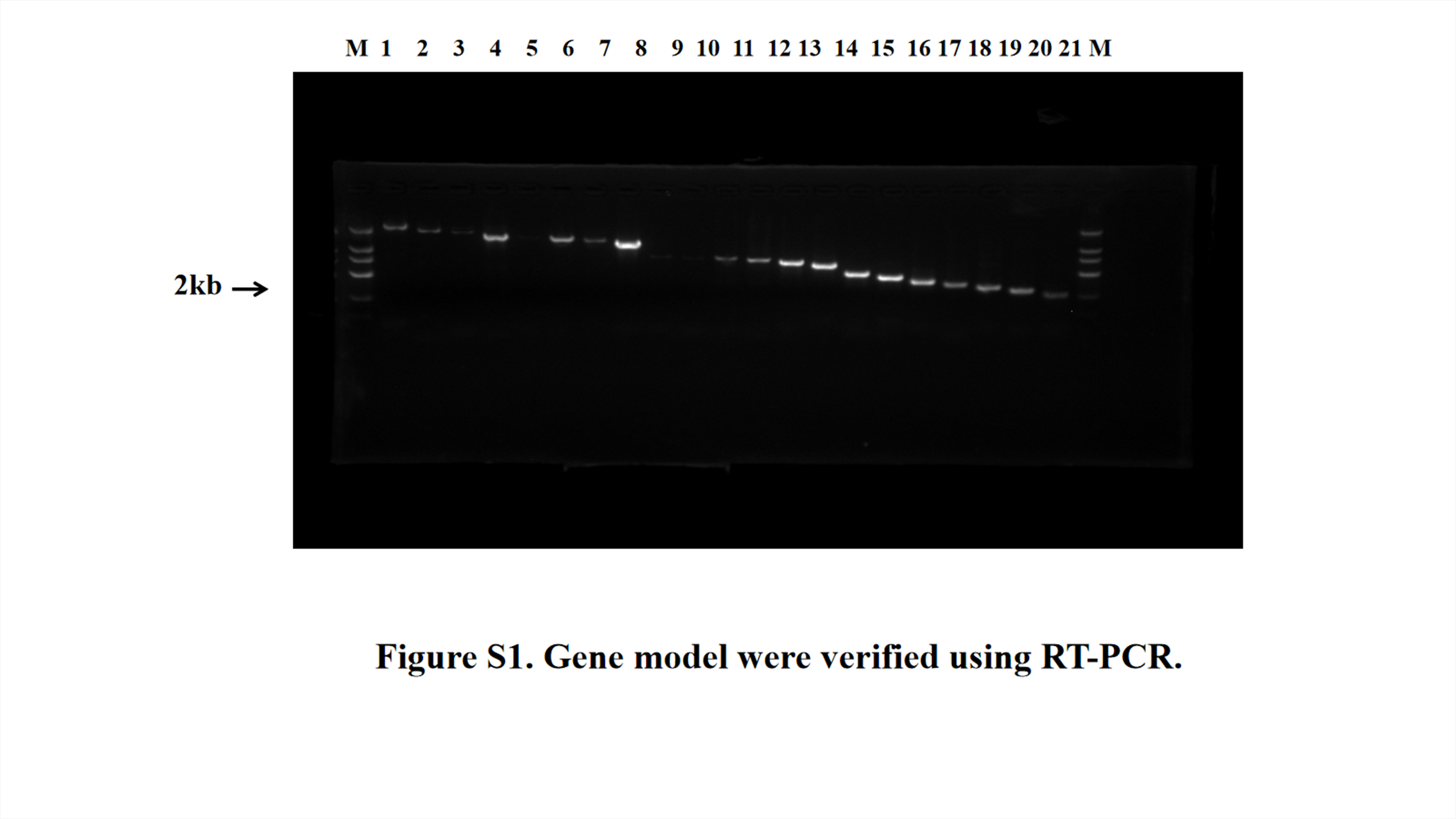

Supplement: Supplementary file 1 [file plants-13-01604-s001.zip › Figure S1.tif]

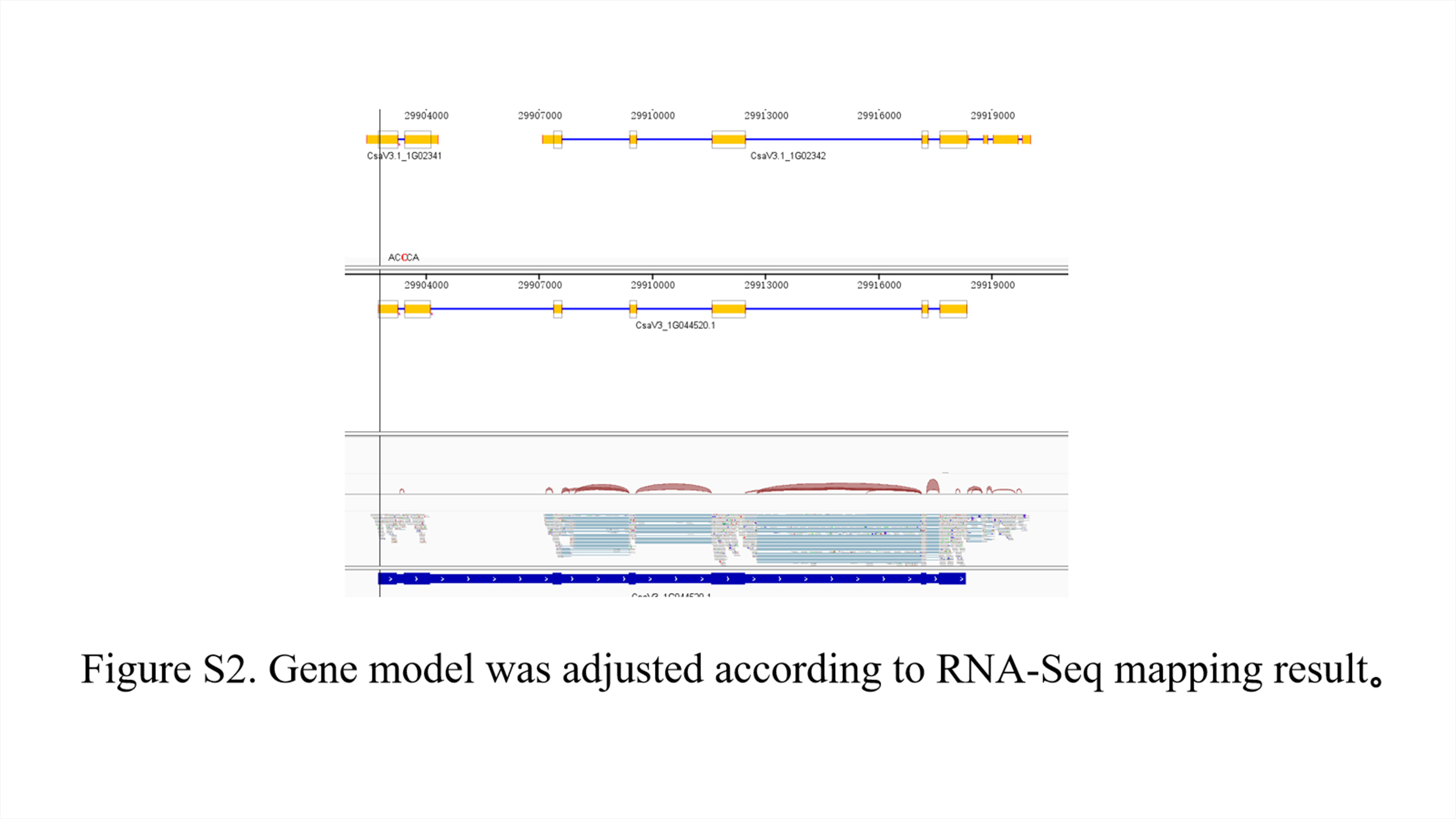

Supplement: Supplementary file 1 [file plants-13-01604-s001.zip › Figure S2.tif]

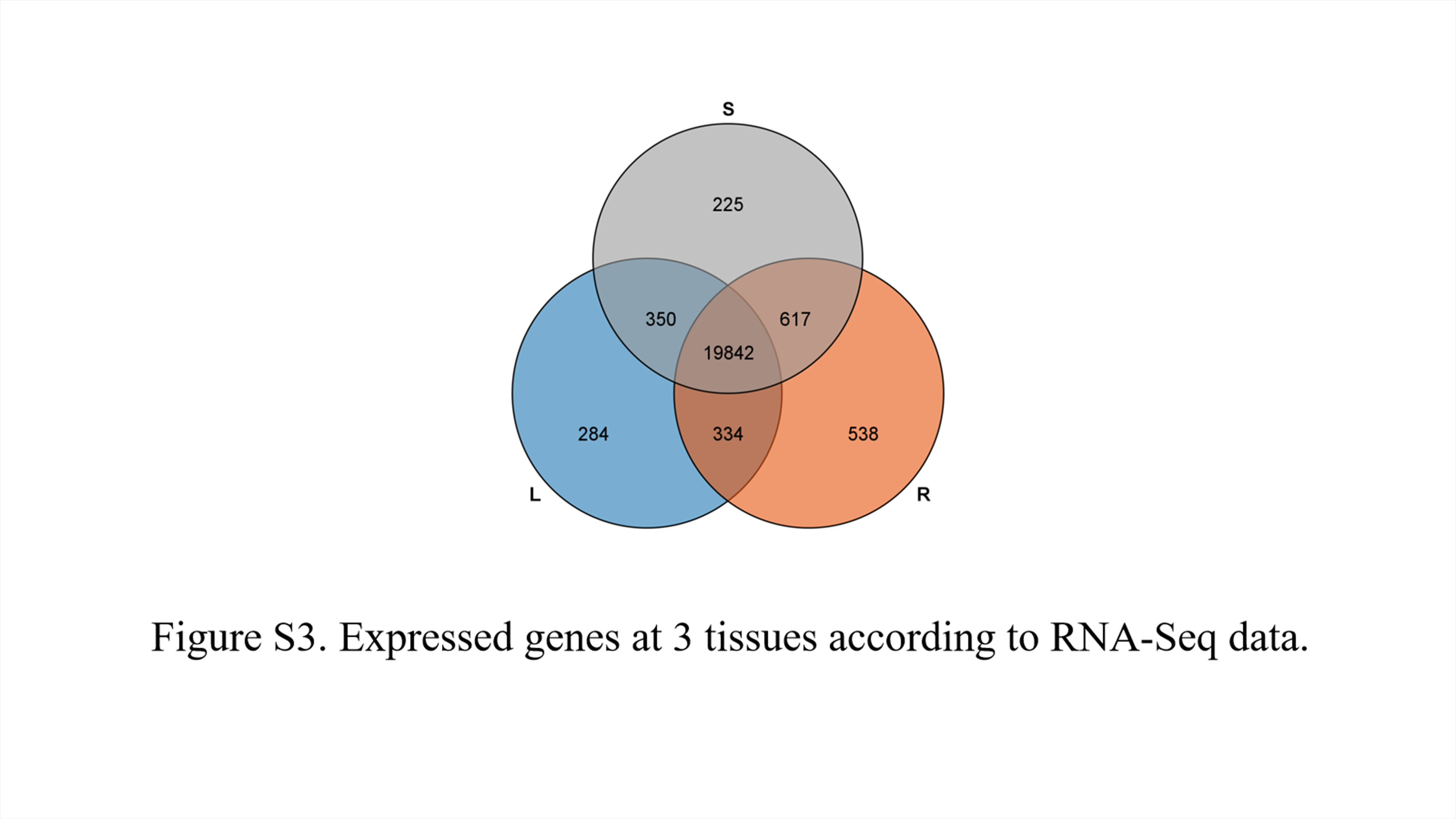

Supplement: Supplementary file 1 [file plants-13-01604-s001.zip › Figure S3.tif]

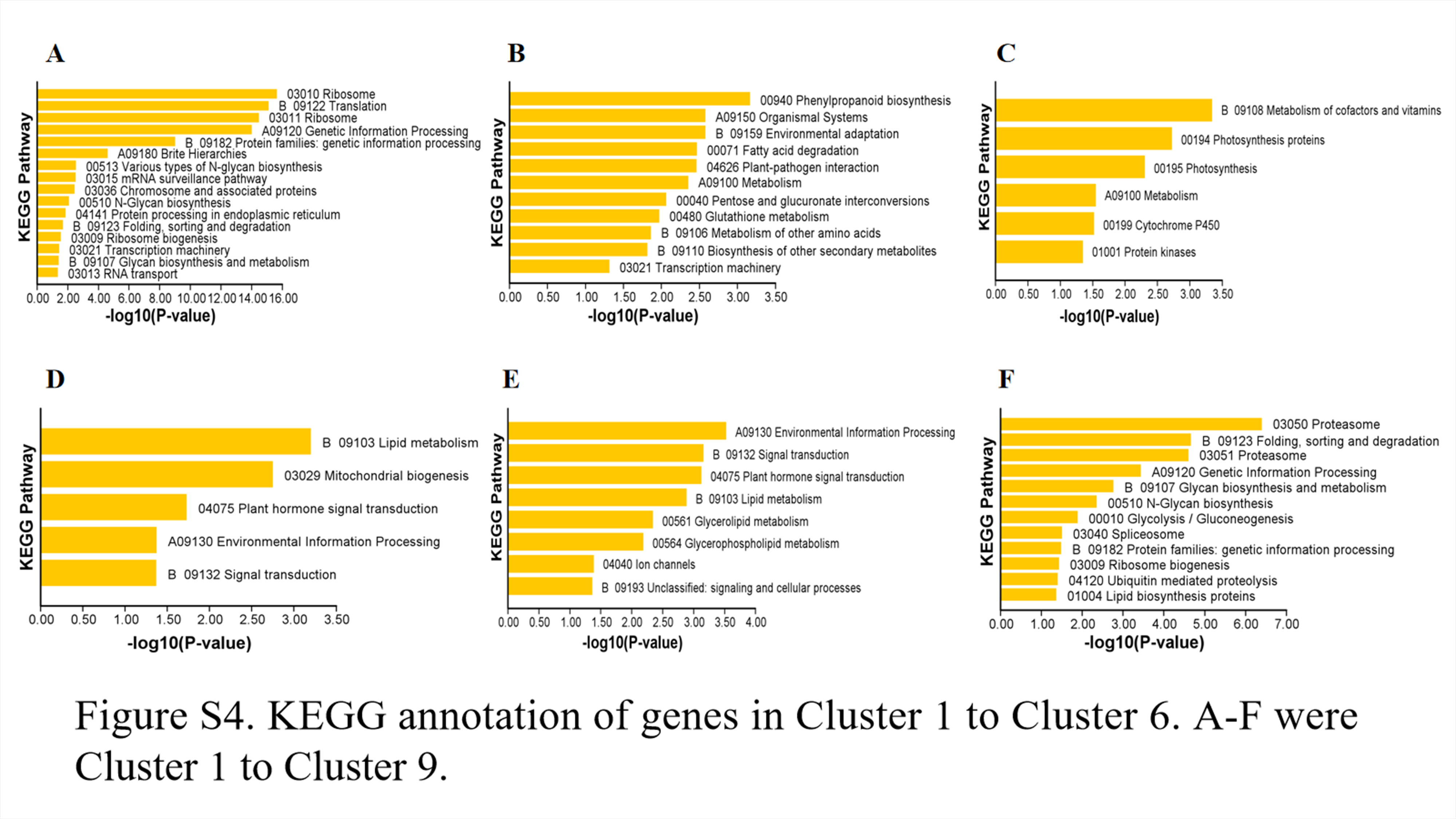

Supplement: Supplementary file 1 [file plants-13-01604-s001.zip › Figure S4.tif]

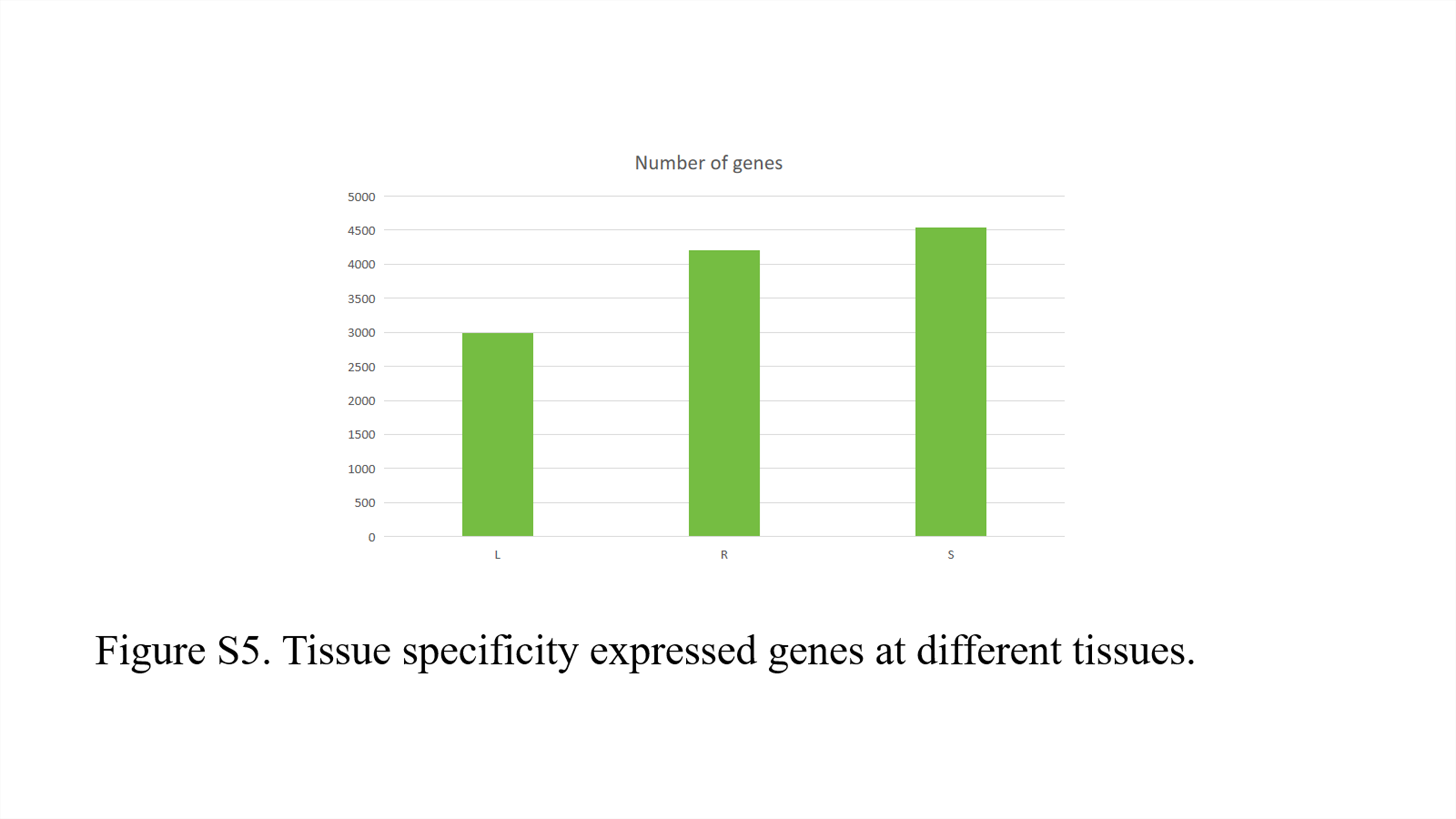

Supplement: Supplementary file 1 [file plants-13-01604-s001.zip › Figure S5.tif]
